# Vitiligo Possibly Triggered by COVID-19 Vaccination

**DOI:** 10.7759/cureus.20902

**Published:** 2022-01-03

**Authors:** Michelle Militello, Austin B Ambur, William Steffes

**Affiliations:** 1 Dermatology, Rocky Vista University College of Osteopathic Medicine, Denver, USA; 2 Dermatology, Kansas City University, Oveido, USA; 3 Dermatology, Advanced Dermatology and Cosmetic Surgery, Windemere, USA

**Keywords:** autoimmunity, autoimmune, vitiligo, vaccination, covid-19

## Abstract

Several cutaneous manifestations following COVID-19 vaccination have been cited in the literature since the beginning of the pandemic. Two case reports regarding the development of vitiligo after receiving the COVID-19 vaccine. Herein, we present a case report of a patient who developed new-onset vitiligo two weeks after receiving her COVID-19 vaccine. Although the pathogenesis is unclear, it may be related to the inflammatory cells involved in both the pathogenesis of vitiligo and the mechanism by which the COVID-19 vaccine stimulates the immune system. This case report highlights the need for further investigation into the link between COVID-19 and the development of vitiligo.

## Introduction

Since the start of the coronavirus (COVID-19) pandemic, there have been several cutaneous manifestations in COVID-19 vaccinated patients cited in the literature. Most commonly are injection site reactions, as well as localized facial swelling in areas where previous dermal fillers have been placed [1]. To our knowledge, there have only been two case reports regarding the development of vitiligo after receiving the COVID-19 vaccine. Herein, we present a case report of a patient who developed new-onset vitiligo just two weeks after receiving her COVID-19 vaccine.

## Case presentation

**Figure 1:**
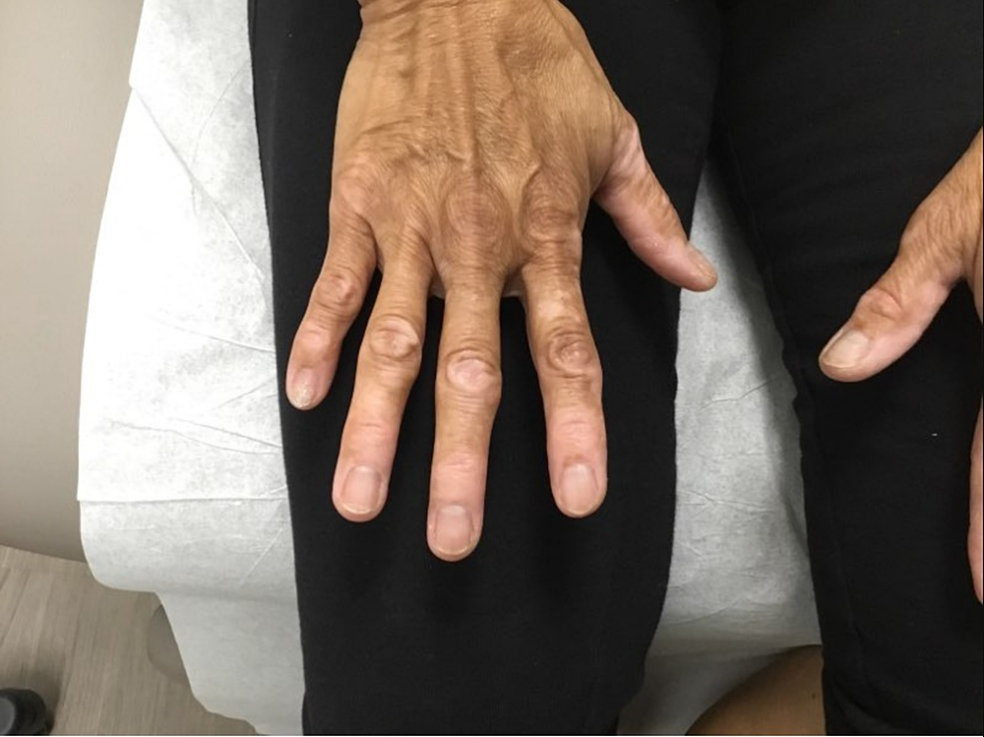
Multiple depigmented patches located on the bilateral dorsal hands

A 67-year-old woman with no significant past medical history presented to the dermatology clinic for an evaluation of a new onset of skin discoloration of the bilateral hands which had been present for several months. The patient reported that the discoloration began two weeks after receiving the COVID-19 Moderna vaccine. Physical examination revealed multiple depigmented patches primarily located on the bilateral dorsal hands (Figure 1). The patches were consistent with vitiligo and were confirmed under Woods lamp examination. Her past medical history was unremarkable for thyroid disease, pernicious anemia, or other autoimmune diseases. Medication reconciliation was unremarkable for any systemic or topical therapy reported to be associated with drug-induced vitiligo. COVID-19 vaccination was determined to be the most likely culprit for the vitiliginous skin changes given that the lesions occurred two weeks after the vaccine and the lack of additional risk factors. She was treated with fluocinonide 0.05% topical cream applied twice daily.

## Discussion

Although our patient's vitiligo may have developed idiopathically, the temporal association of her presentation after the vaccine combined with the absence of other risk factors makes it probable that her vitiligo was likely secondary to her COVID-19 vaccination. There have been two previous case reports that have also indicated a temporal association between the COVID-19 vaccine and the development of new-onset vitiligo; one occurring in a 58-year-old man with a prior history of ulcerative colitis following the Pfizer vaccine and another occurring in a 61-year-old woman following the Moderna vaccine [2,3].

The pathophysiology leading to vitiligo in the setting of COVID-19 vaccination remains unclear but there are several hypotheses. Most notably, there may be a link between the inflammatory cells involved in each pathway. Immune mechanisms such as antigen presentation, cytokine production, epitope spreading, and polyclonal activation of B cells are involved in both the anti-infectious immune response as well as in autoreactivity [4]. In the case of vitiligo, Kaminetsky and Rudikoff have postulated that specifically, melanocytes may become unintentional targets for antibodies and immune cells newly produced by the vaccine [2]. Given that the development of vitiligo involves the destruction of melanocytes by autoreactive CD8+ T cells and successful vaccination also involves an extensive CD8+ T-cell response, this hypothesis may be plausible [5].

COVID-19 is still a relatively new disease and the vaccine has only been available since December 2020; therefore, little is known regarding the possibility of triggering an autoimmune disease post-vaccination. However, there have been other reports of patients developing autoimmune conditions, such as immune thrombocytopenic purpura after receiving the vaccine [6].

## Conclusions

Given that the COVID-19 vaccination has the potential to cause an extensive inflammatory response, it would be unsurprising if more cases of autoimmunity begin to emerge. Our understanding of autoimmune reactions following COVID-19 vaccination continues to expand, and this case emphasizes that COVID-19 vaccination may be among the list of triggers for vitiligo. This case report along with the two other case reports highlights the need for future studies to further investigate this link.

## References

[1] Rice SM, Ferree SD, Mesinkovska NA, Kourosh AS (2021). The art of prevention: COVID-19 vaccine preparedness for the dermatologist. Int J Womens Dermatol.

[2] Kaminetsky J, Rudikoff D (2021). New-onset vitiligo following mRNA-1273 (Moderna) COVID-19 vaccination. Clin Case Rep.

[3] Aktas H, Ertuğrul G (2021). Vitiligo in a COVID-19-vaccinated patient with ulcerative colitis: coincidence? [PREPRINT]. Clin Exp Dermatol.

[4] Velikova T, Georgiev T (2021). SARS-CoV-2 vaccines and autoimmune diseases amidst the COVID-19 crisis. Rheumatol Int.

[5] Riding RL, Harris JE (2019). The role of memory CD8+ T cells in vitiligo. J Immunol.

[6] Malayala SV, Mohan G, Vasireddy D, Atluri P (2021). Purpuric rash and thrombocytopenia after the mRNA-1273 (Moderna) COVID-19 vaccine. Cureus.

